# Application of DNA barcodes in the genetic diversity of hard ticks (Acari: Ixodidae) in Kazakhstan

**DOI:** 10.1007/s10493-023-00893-1

**Published:** 2024-02-22

**Authors:** Ziwei Zheng, Weixin Zeng, Suwen Wang, Wenbo Tan, Xiaobo Lu, Kenesbay Kairullayev, Ligu Mi, Wurelihazi Hazihan, Gang Liu, Meihua Yang, Yuanzhi Wang

**Affiliations:** 1 Department of Basic Medicine, School of Medicine, Shihezi University, Shihezi City, Xinjiang Uygur Autonomous Region 832002 People’s Republic of China; 2 College of Animal Science and Technology, Shihezi University, Shihezi, Xinjiang Uygur Autonomous Region 832002 People’s Republic of China; 3 The First Affiliated Hospital, School of Medicine, Shihezi University, Shihezi City, Xinjiang Uygur Autonomous Region 832002 People’s Republic of China; 4 Biological sciences, Department of Food Engineering, Kazakh National Agrarian University, Almaty Province, Republic of Kazakhstan; 5 Department of Forest, College of Agriculture, Shihezi University, Shihezi City, Xinjiang Uygur Autonomous Region 832003 People’s Republic of China

**Keywords:** Kazakhstan, DNA barcodes, Genetic diversity, Hard ticks

## Abstract

Forty-five tick species have been recorded in Kazakhstan. However, their genetic diversity and evolutionary relationships, particularly when compared to ticks in neighbouring countries, remain unclear. In the present study, 148 mitochondrial *cytochrome c oxidase subunit I* (*COI*) sequence data from our laboratory and NCBI (National Center for Biotechnology Information; https://www.ncbi.nlm.nih.gov/) data were used to address this knowledge gap. Phylogenetic analyses showed that *i*) *Hyalomma anatolicum anatolicum* (Koch, 1844) ticks from Jambyl Oblast (southeastern Kazakhstan) and Gansu Province (northwestern China) constituted a newly deviated clade; and *ii*) *Dermacentor reticulatus* (Fabricius, 1974) ticks from South Kazakhstan Oblast were closer to those in Romania and Turkey. The network diagram of haplotypes showed that *i*) the H-1 and H-2 haplotypes of *Dermacentor marginatus* (Sulzer, 1776) ticks from Zhetisu and Almaty were all newly evolved; and *ii*) the H-3 haplotypes of *Haemaphysalis erinacei* (Pavesi, 1884) from Almaty Oblast and Xinjiang Uygur Autonomous Region (northwestern China) were evolved from the H-1 haplotype from Italy. In the future, more *COI* data from different tick species, especially from Kazakhstan and neighbouring countries, should be employed in the field of tick DNA barcoding.

**Supplementary Information:**

The online version contains supplementary material available at 10.1007/s10493-023-00893-1.

## Introduction

Hard ticks are ectoparasites of terrestrial vertebrates that require a different host for each developmental stage, namely the larval, nymph, and adult stages (Leal et al. 2020). Host activity, such as the seasonal migration of migratory birds and the international livestock trade, plays an important role in tick dissemination (Tsao et al. 2021).

Kazakhstan, with an area of 2,724,900 square kilometers (Yang et al. 2002), is the ninth-largest country in the world, and is adjacent to Russia, Turkmenistan, Uzbekistan and China. To date, 45 tick species, belonging to seven genera including *Ixodes*, *Hyalomma*, *Dermacentor*, *Rhipicephalus*, *Haemaphysalis*, *Argas* and *Ornithodoros*, have been reported in Kazakhstan (Perfilyeva et al. 2020).

Mitochondrial genes, such as *12 S rDNA*, *16 S rDNA*, *cytochrome c oxidase subunit I* (*COI*), and *COII*, are the most common molecular markers used to identify tick species (Murrell et al. 2000; Leo et al. 2010). The mitochondrial *COI*, as the standard for DNA barcoding, has played an important role in intra-/inter-species identification and the study of genetic diversity (Hebert et al. 2003). However, the biology of ticks plays a decisive role in the epidemic characteristics of tick-borne diseases in different regions, and limited studies have been conducted in Kazakhstan using DNA barcodes to analyze the genetic diversity and evolutionary relationships of ticks, especially compared to its neighbouring countries. To address this knowledge gap, this study conducted phylogenetic analyses based on *COI* sequences to explore intra-/inter-species tick evolution in central Asia.

## Materials and methods

### Tick sampling and morphological identification

**Table 1:** Tick collection data during 2016–2023 in Kazakhstan

Year	Oblast	Host	Hard/ soft tick	Number
2016–2019	Almaty	Cattle, sheep, hedgehog, off-host	Hard tick	596
2018–2019	East Kazakhstan	Cattle, sheep	Hard tick	1625
2018–2019	Jambyl	Cattle, sheep	Hard tick	752
2018–2019, 2023	Kyzylorda	Camel, cattle, sheep,	Hard tick	4693
2018–2019, 2023	South Kazakhstan	Cattle, chicken, sheep, dog, off-host	Hard tick	3886
2018–2019	South Kazakhstan	Chicken, off-host	Soft tick	1472
2023	Zhetisu	Cattle	Hard tick	71
**Total**				13,095

Under the cooperation agreement between Shihezi University and Kazakh National Agrarian University, a total of 13,095 nymphs and adult ticks were collected in Kazakhstan during 2016–2023. Parasitizing ticks were collected from the entire body of each animal, including camel (*Camelus bactrianus*), cattle (*Bos taurus*), chicken (*Gallus gallus f. domestica*), dog (*Canis lupus familiaris*), hedgehog (*Hemiechinus auritus*), horse (*Equus ferus caballus*), and sheep (*Ovis aries*) (Horak et al. 2011; Wang et al. 2015). Off-host ticks were collected using the dragging-flagging method and by directly capturing ticks from the ground (Tsunoda et al. 2004; Zhang et al. 2016). The sampling information is shown in Table 1. All ticks were placed in tubes with 75% ethanol, stored at − 20 °C and morphologically identified according to standard taxonomic keys (Walker et al. 2003).

### DNA extraction, polymerase chain reaction (PCR) amplification, and sequencing

After morphological identification, 464 representative tick specimens, with three to 10 ticks for each tick species obtained at each sampling site, were used to analyze the genetic diversity. DNA was extracted from representative ticks using TIANamp Genomic DNA Kit (TIANGEN, Beijing, China) according to the manufacturer’ s instructions. About 710-bp fragments of the *COI* genes were amplified via PCR. The primers and PCR cycling conditions are shown in Appendix Table 1. The newly generated sequences of *COI* were manually edited, aligned, and compared to the reference GenBank (National Center for Biotechnology Information, NCBI) sequences using the nucleotide BLASTN program (https://blast.ncbi.nlm.nih.gov) (Hornok et al. 2016). A total of 38 *COI* sequences of tick samples were deposited in the NCBI GenBank (accession No. MN907836, MN907834, MN907826, MN907832, MN964336, MN907838, MN964340, MN907848, MT079206, MN689420, MN689425, MN689410, MN907846, MN841463, MN689436, MN689429, MN689434, MN907845, MN689404, MN964337, MN964342, MN821375, MN964341, MN907835, MN853164, MN853163, OR533607, OR533660 and OR533790-OR533799).

### Sequence analyses

The data obtained in the laboratory were combined with the NCBI (https://www.ncbi.nlm.nih.gov/) data retrieved on March 27, 2023. The above data were resampled 1,000 times to generate bootstrap values. Phylogenetic relationships were inferred using the maximum likelihood (ML) method. Evolutionary analyses were conducted in MEGA X (shown in Appendix Fig. 1). The genetic diversity was estimated using the haplotype (h), haplotype diversity (Hd) and nucleotide diversity (Pi) indices with the DNAsp ver. 5.10.01 program. Median-joining (MJ) networks were generated with the Network ver. 10.2.0 software to display the configuration of haplotypes (Wetjen et al. 2020).

## Results and discussion

**Fig. 1:**
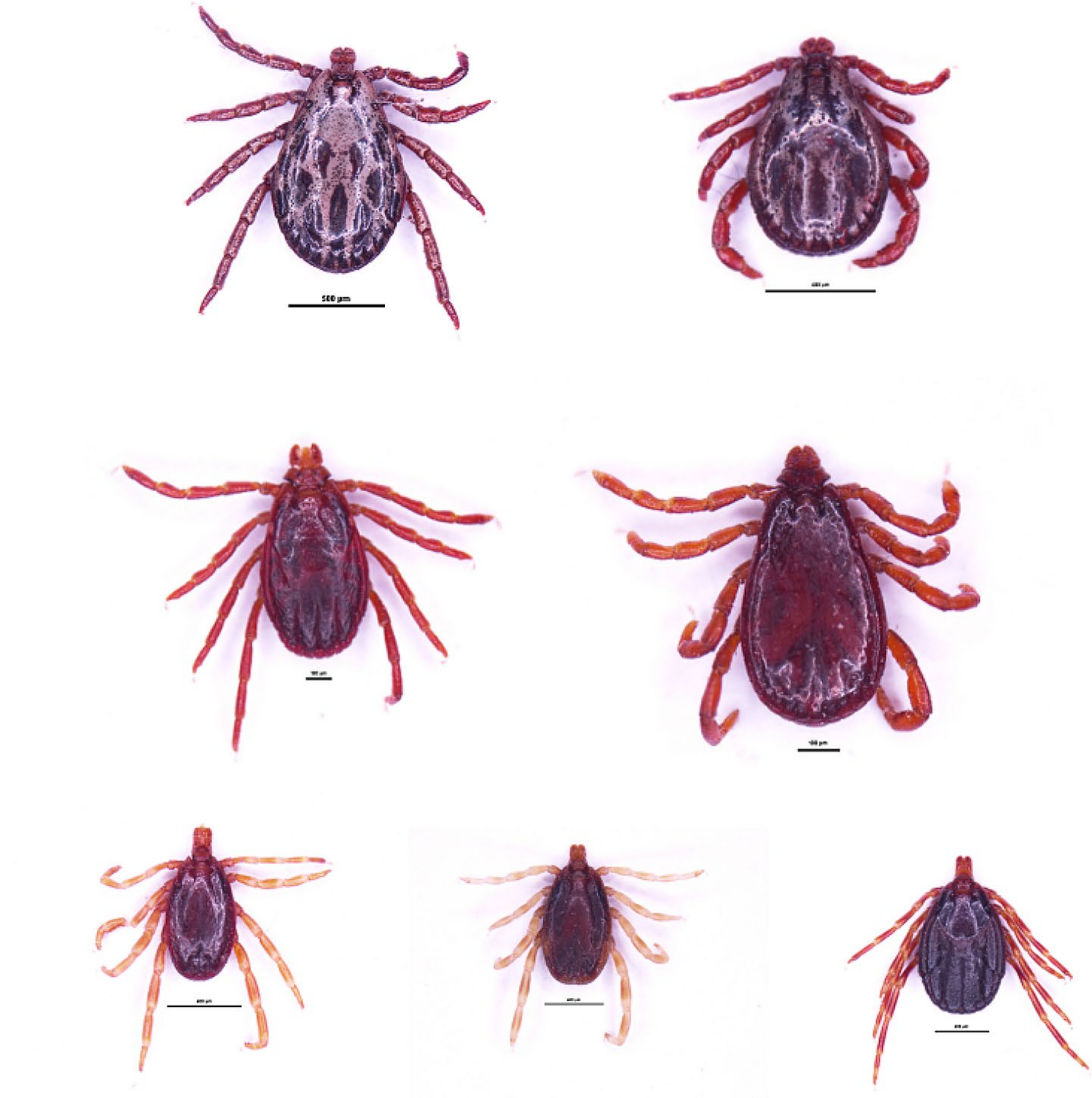
Morphological tick identification results

Morphological and molecular identification confirmed that seven hard tick species were identified, namely *Dermacentor marginatus*, *Dermacentor reticulatus*, *Rhipicephalus sanguineus* (Latreille, 1806), *Rhipicephalus turanicus* (Pomerantzev, 1940), *Hyalomma anatolicum*, *Hyalomma asiaticum asiaticum* (Schulze et Schlottke, 1929) and *Hyalomma scupense* (Schulze, 1918). Photographs of them in turn are shown in Fig. 1.

The analysis of the *COI* phylogenetic tree showed that *i*) *Hy*. *anatolicum* ticks (accession Nos. MN841463 and MH459382) from Jambyl Oblast and Gansu Province (northwestern China) constituted a newly deviated clade, while the corresponding species from Khorasan-e Razavi Province (northeastern Iran) (KP219867) and Turkey (MT230046), were ancestor populations; *ii*) *D*. *reticulatus* ticks (OR533790) from South Kazakhstan Oblast were more closely related to those in Romania (KT87452) and Turkey (KT877453); and *iii*) *D*. *marginatus* ticks from Kazakhstan had two clades. One clade (MN907848) from Almaty Oblast was closer to that (FN394327) from Balint (Romania), and another clade (MN907836) from East Kazakhstan Oblast was closer to that (MN517831) from Xinjiang Uygur Autonomous Region (XUAR, northwestern China). Previously, *H. erinacei* was classified into three subspecies, including *H. e. erinacei* (distribution in North Africa and southern Europe, in particular in Spain, Italy and the western Balkans) (Tovornik and Cerný 1974), *H. e. taurica* (distributed in the Middle East and the eastern Balkans) (Zlatanova 1991) and *H. e. turanica* (distributed in Central Asia) (Hornok et al. 2016). In this study, *H. erinacei* (accession No. MN841464) from Almaty Oblast was clustered into *H. e. turanica*, together with the corresponding sequence (KU880621) from XUAR (northwestern China), rather than those from Europe, including Tokat Province (Turkey) (KX901844), Romania (KU885986), and Italy (KX237631). The *COI* BLAST analysis showed that the sequence from Kazakhstan shared 100% identity with that from China (KU880621), but had only 95.13%, 95.10% and 94.81% identities with those from Turkey (KX901844), Romania (KU885986) and Italy (KX237631), respectively. This finding was consistent with the analysis of the *16 S rDNA* phylogenetic tree (Hornok et al. 2016).

**Fig. 2:**
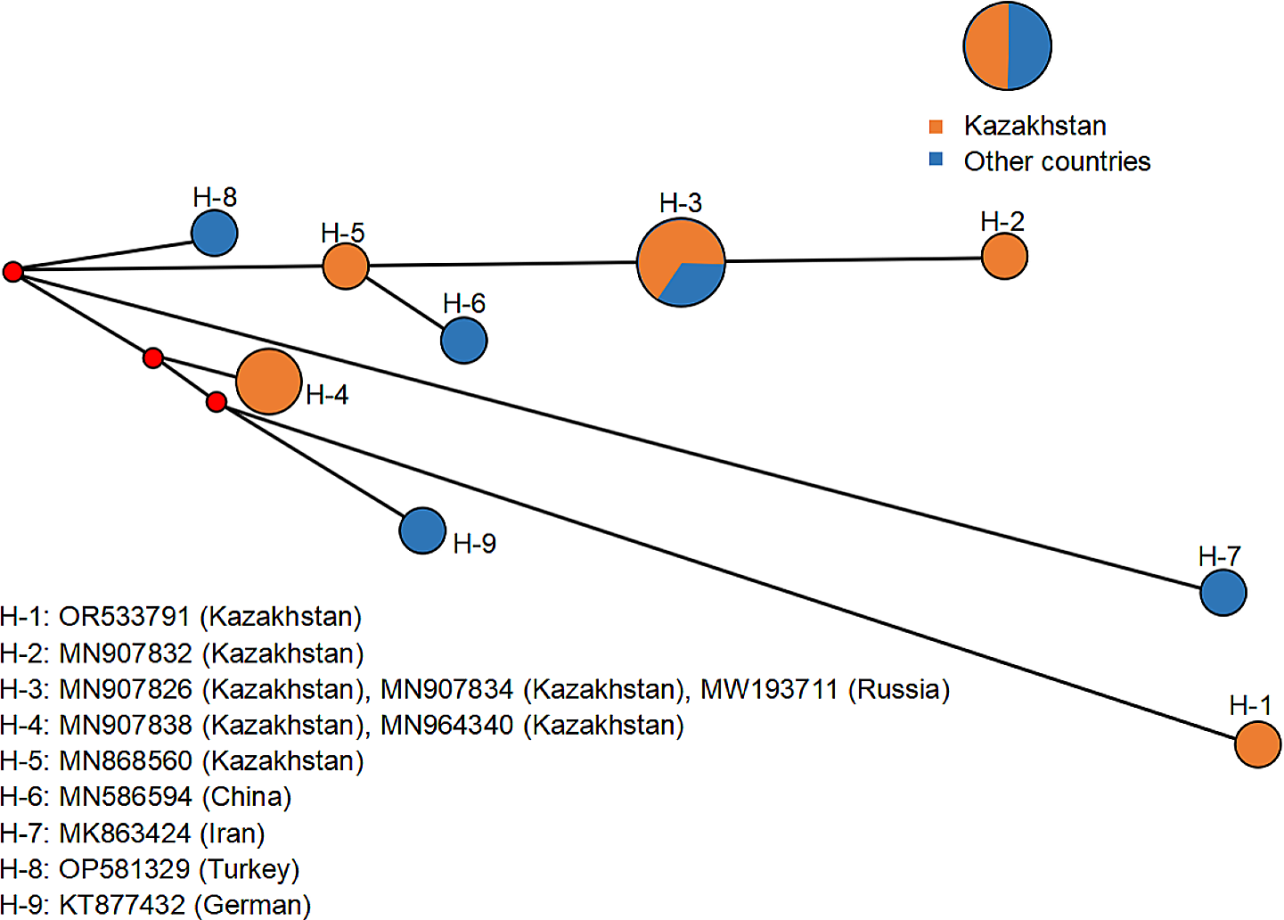
Network diagram of *Dermacentor marginatus* haplotypes

**Fig. 3:**
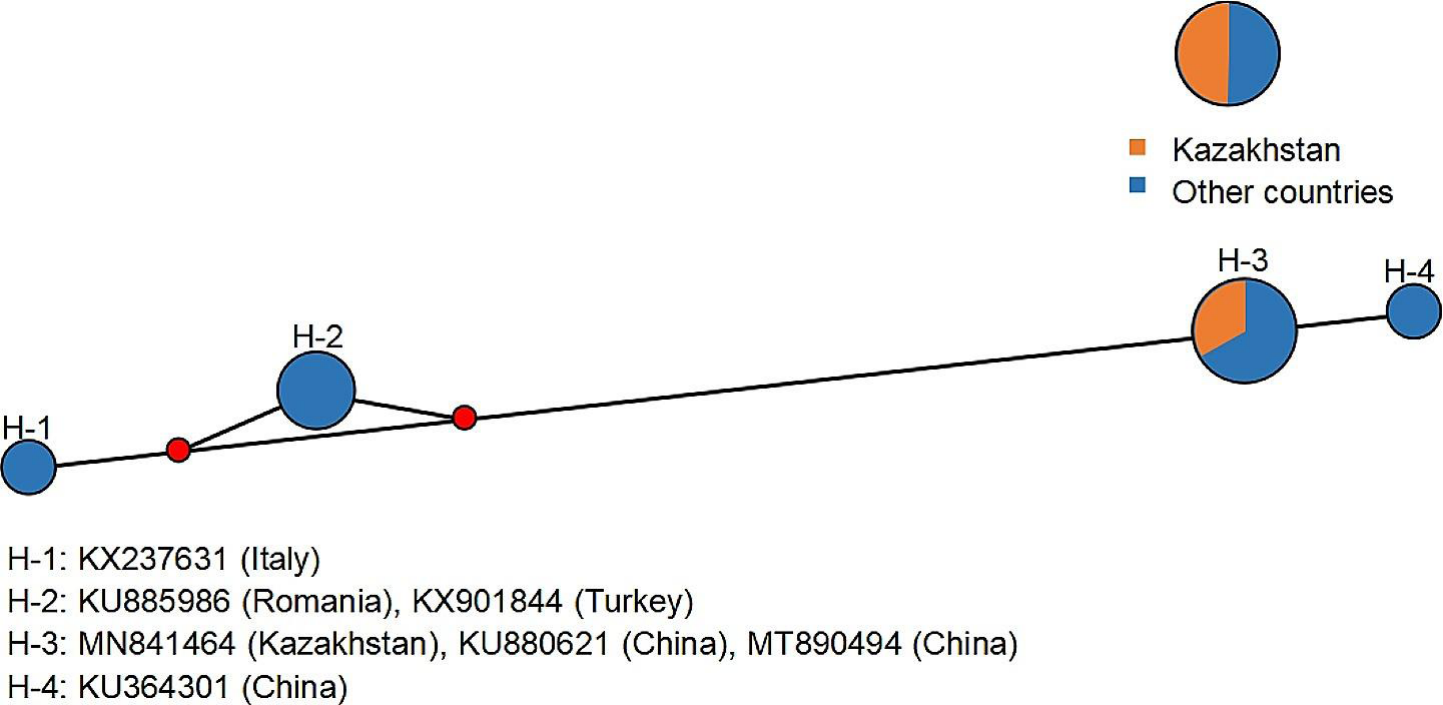
Network diagram of *Haemaphysalis erinacei* haplotypes

The network diagram based on the *COI* in the haplotypes was shown as follows. Firstly, *D*. *marginatus* ticks were highly divergent, and nine haplotypes were found. The haplotype diversity was 0.9394, while the nucleotide diversity was 0.02101. The H-3 haplotype was the most dominant. The H-1 and H-2 haplotypes from Zhetisu Oblast and Almaty Oblast, the H-6 haplotype from XUAR (northwestern China), the H-7 haplotype from Iran, the H-8 haplotype from Turkey and the H-9 haplotype from Germany were all newly evolved. More interestingly, *D*. *marginatus* ticks (belonging to the H-3 and 6 haplotypes) in Russia and XUAR (northwestern China), respectively, were evolved from the H-5 haplotype from South Kazakhstan Oblast (shown in Fig. 2). This result might be related to the close geographical distance between southeast Kazakhstan, northwestern China and the far east of Russia. Secondly, *H*. *erinacei* harbored four haplotypes. The haplotype diversity was 0.8095, and the nucleotide diversity was 0.02972. The H-3 haplotypes from Almaty Oblast (MN841464) and XUAR (northwestern China) (KU880621 and MT890494) were evolved from the H-1 haplotype from Italy (KX237631) (shown in Fig. 3). Although there is limited *COI* data available around the world, the ancestor of *H. erinacei* may have originated in Europe and developed morphological and molecular differences with crustal movement and host migration.

Central Asia covers about 4 million km^2^, exhibits many shared characteristics in terms of climate, wildlife hosts and geographical habitats. In total, 148 *COI* sequences were involved in the current study. In future work, more *COI* data from different tick species, especially data from Kazakhstan and its neighbouring countries, will be employed. This will allow for the more systematic exploration of intra-/inter-species tick evolution in central Asia.

## Conclusions

This article describes the genetic diversity and evolutionary relationships of hard ticks in Kazakhstan based on the analysis of *COI* data. *Hy*. *anatolicum* ticks from Jambyl Oblast and Gansu Province (northwestern China) constituted a new population. *D*. *reticulatus* ticks from South Kazakhstan Oblast were closer to those in Romania and Turkey. The H-1 and H-2 haplotypes of *D*. *marginatus* ticks from Zhetisu Oblast and Almaty Oblast were newly evolved. The H-3 haplotypes of *H*. *erinacei* from Almaty Oblast and XUAR (northwestern China) were evolved from the H-1 haplotype from Italy.

### Electronic supplementary material

Below is the link to the electronic supplementary material.

Supplementary Material 1

Supplementary Material 2

## Data Availability

The datasets generated during the current study are available in the GenBank repository, http://www.ncbi.nlm.nih.gov/genbank/.
