# Supplementary material for: Application of DNA barcodes in the genetic diversity of hard ticks (Acari: Ixodidae) in Kazakhstan
Source: Exp Appl Acarol. 2024 Feb 22;92(3):547–54. doi: 10.1007/s10493-023-00893-1 (PMC11035449; doi:10.1007/s10493-023-00893-1)
Supplement: Supplementary file 1 — Supplementary Material 1 [file 10493_2023_893_MOESM1_ESM.docx]

**Appendix Table 1** PCR protocol for the detection of 464 representative tick

specimens, Kazakhstan.

The PCR equipment was a Mastercycler X50s thermal cycler, Eppendorf, Hamburg, Germany.

1. PCR amplification of *COI* mitochondrial gene sequences from 464 representative tick specimens

Each reaction consisted of 1 μL of tick genomic DNA (50 ng) and 25 μL of a PCR mix containing 16.5 μL of ultrapure water, 2.4 μL of 10× PCR buffer, 2 μL of dNTPs (10 mmol/L), 1.5 μL of the DNA template (50ng/μL), 1 μL of each primer (20 μmol/L) and 0.6 μL of Taq DNA polymerase (Taq DNA Polymerase, GDSBio, Guangzhou, China). The cycling conditions consisted of an initial 5-min denaturation at 94°C, followed by 37 cycles at 94°C for 30 s, 46°C for 30 s, and 72°C for 90 s, with a final extension at 72°C for 8 min.

Nucleotide sequences of the primers used for the identification of ticks

| Gene | Primer | Sequence（5' to 3'） | Reference |
| --- | --- | --- | --- |
| *COI* | LCO1490 | GGT CAA CAA ATC ATA AAG ATA TTG G |  |
|  | HCO2198 | TAA ACT TCA GGG TGA CCA AAA AAT CA |  |

References

Folmer O, Black M, Hoeh W, Lutz R, Vrijenhoek R (1994) DNA primers for amplifification of mitochondrial *cytochrome c oxidase subunit I* from diverse metazoan invertebrates. Mol Mar Biol Biotechnol 3(5):294-299
