# Supplementary material for: Application of DNA barcodes in the genetic diversity of hard ticks (Acari: Ixodidae) in Kazakhstan
Source: Exp Appl Acarol. 2024 Feb 22;92(3):547–54. doi: 10.1007/s10493-023-00893-1 (PMC11035449; doi:10.1007/s10493-023-00893-1)

**Appendix Fig 1:** Maximum-likelihood (ML; 1,000 bootstrap replicates) phylogenetic tree based on the *COI* constructed with MEGA X, using the sequences of tick species from Kazakhstan (▲) in this study and the sequences available in the GenBank.


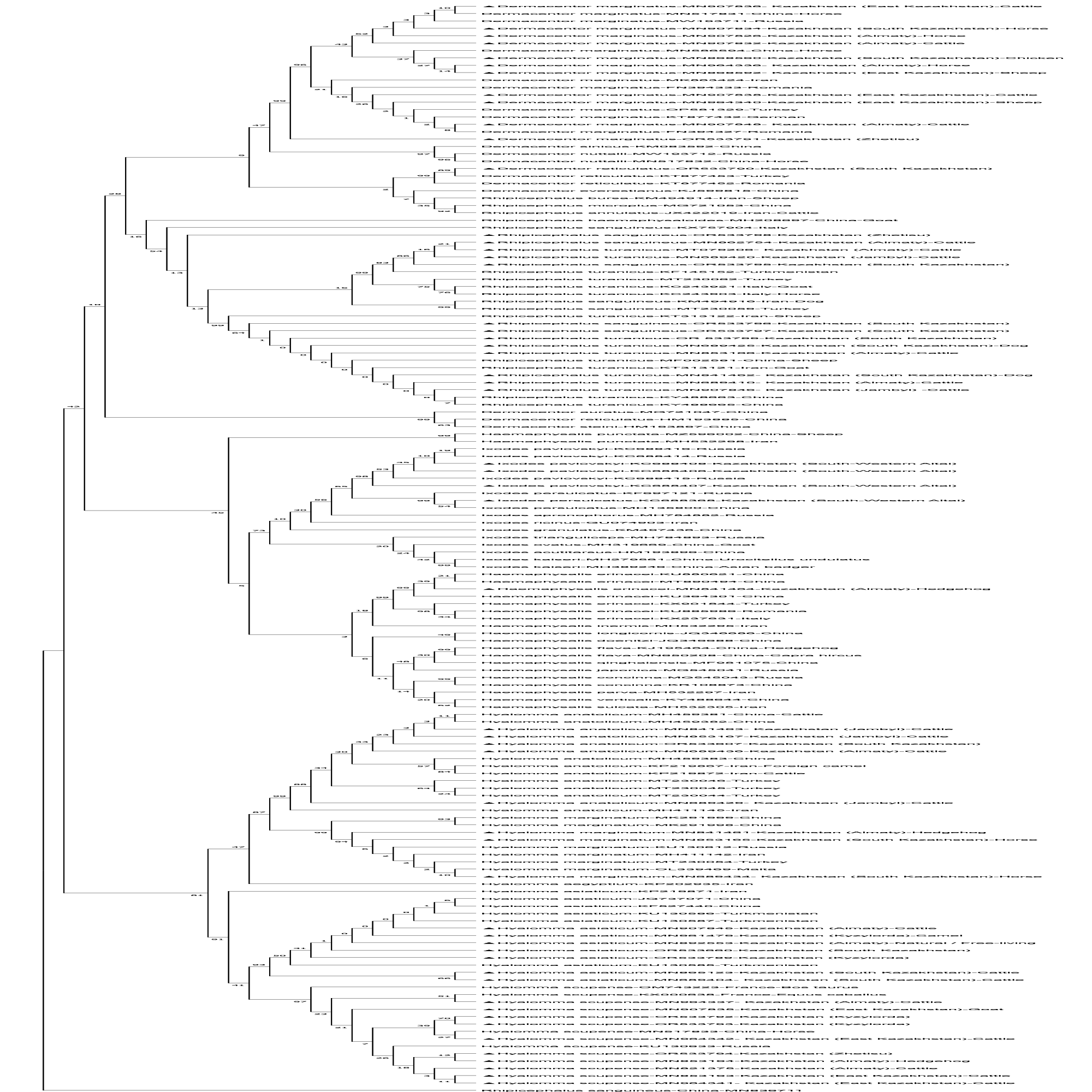

Supplement: Supplementary file 2 — Supplementary Material 2 [file 10493_2023_893_MOESM2_ESM.docx]
